# *Schinus terebinthifolia* Raddi—Untargeted Metabolomics Approach to Investigate the Chemical Variation in Volatile and Non-Volatile Compounds

**DOI:** 10.3390/metabo14110612

**Published:** 2024-11-11

**Authors:** Mara Junqueira Carneiro, Guilherme Perez Pinheiro, Elisa Ribeiro Miranda Antunes, Leandro Wang Hantao, Thomas Moritz, Alexandra Christine Helena Frankland Sawaya

**Affiliations:** 1 LabMetaMass, Faculty of Pharmaceutical Science, State University of Campinas, Campinas 13083-871, SP, Brazil; marajunqueira@gmail.com; 2 Institute of Biology, State University of Campinas, Campinas 13083-871, SP, Brazil; pinheiro.gperez@gmail.com (G.P.P.);; 3 Institute of Chemistry, State University of Campinas, Campinas 13083-871, SP, Brazil; wang@unicamp.br; 4 Department of Forest Genetics and Plant Physiology, Swedish University of Agricultural Sciences, 750 07 Umeå, Sweden; 5 Novo Nordisk Foundation Center for Basic Metabolic Research, University of Copenhagen, 1353 Copenhagen, Denmark

**Keywords:** pink pepper, *Aroeira*, UHPLC-MS, GC-MS, seasonality, plant sex

## Abstract

Context: *Schinus terebinthifolia* Raddi is used in Brazilian folk medicine due to the wound healing and antiseptic properties of its bark, and its fruit are used as a condiment. However, the aerial parts of this plant have been studied and present some bioactive compounds as well. Objectives: The aim of this study was to investigate the variation in volatile and non-volatile composition of *S. terebinthifolia* leaves using untargeted metabolomics. Material and Methods: The leaves of four trees were collected over one year; ethanolic extracts were analyzed by UHPLC-MS and fresh leaves were analyzed by GC-MS using HS-SPME. The data were processed using online software. Results: The results suggest seasonality interfered little with the chemical composition of leaves. On the other hand, the sex of the plant clearly determined the chemical composition of both volatile and non-volatile compounds. Discussion and conclusions: Chemical variability between plants with male and female flowers is fundamental information for the standardized use of its leaves. Compounds with important biological activities were putatively identified, confirming the potential use of *S. terebinthifolia* leaves as a source of bioactive compounds, reducing waste and increasing economic gains for local farmers throughout the year.

## 1. Introduction

*Schinus terebinthifolia* Raddi belongs to the Anacardiaceae family and is popularly known as pink pepper tree and as *aroeira* in Brazil. It is native to South America, amply distributed in Brazil, and successfully introduced in Central America, some parts of the United States, Africa, Asia, and Mediterranean Europe [1]. The female flowers of this dioecious species produce red drupes known as pink pepper [2] with a slightly peppery flavor and are appreciated as a condiment throughout many countries [3]. They present a source of income for local communities although fruit is only present during a specific period of the year and only in female trees.

Studies with non-volatile components in pink pepper extracts were undertaken, showing anti-inflammatory potential [4] related to phenols, anthocyanins, terpenes, and other compounds from the secondary metabolism, which are used as defense mechanisms by the plant [5]. These phenolic compounds not only protect the plants, but also protect the human body from oxidative stress when consumed in food, and may also be used as a natural antioxidant in food systems [5].

Similarly, essential oil in the pink peppers is also involved in their biological effects. The essential oil of mature pink peppers was found to contain 41% β-myrcene and demonstrated antimicrobial activity [6]. Furthermore, all the aerial parts (leaves, flowers, and fruit) contain essential oils with varied antioxidant potential. Male inflorescences (which do not produce fruit) have a high yield of essential oil with strong antioxidant potential [7] indicating a feasible use for the male trees, which are also necessary for fruit production. 

Currently, the commercial value of this species is due to the fruit and, to a lesser extent, its bark. In Brazil, vaginal ovules and gel containing bark extract of *S. terebinthifolia* is provided by the Brazilian Government through the Public Health System [8], although the use of leaves would present a more sustainable source of bioactive molecules. 

Leaves of pink pepper trees are discarded during fruit selection but have shown diverse activities, including anti-tumor activities [9]. The use of *S. terebinthifolia* leaves, from both male and female trees, for the recovery of biologically active substances, presents a viable alternative to reduce waste and increase economic gains for local farmers throughout the year. 

Production of these bioactive compounds depends on endogenous (such as plant age and genes) and exogenous factors (such as temperature, amount of rain, solar radiation, seasonality, circadian rhythm, and physical damage) which influence the secondary metabolism. Previous studies of our group have shown the impact of seasonality and growth conditions on the composition of ethanol extracts [10] and headspace volatiles [11] of other medicinal plants, using both targeted and untargeted metabolomic approaches. 

When proposing the use of any plant species for therapeutic purposes or as a source of components as food additives, it is imperative to map possible causes of variation in its chemical composition in order to collect the material at the most appropriate moment and select the best individuals. Therefore, in this study, four adult trees identified as T1–T4, growing within a two-kilometer radius were selected to investigate the chemical variations in volatile and non-volatile compounds of *S. terebinthifolia*’ leaves over a year, using untargeted metabolomics. Putatively identified compounds were further investigated using Prediction of Activity Spectra for Substances (PASS) software Way2Drug.com © 2011–2024 Version 2.0 to indicate possible activity as in silico tools are often used to suggest possible activities of natural compounds. 

## 2. Materials and Methods

### 2.1. Plant Material

Four adult trees (T1, T2, T3, and T4) were selected within a radius of two kilometers, around the State University of Campinas (UNICAMP), Campinas, SP, Brazil. Vouchers of each individual were deposited at the UNICAMP Herbarium (UEC) numbered UEC 197985; UEC 197983; UEC 197984; and UEC 197333, respectively. This study is registered at the SISGEN website under the code A3B4B86. As all four individuals flowered occasionally, flowers were selected for the determination of their sex toward the end of the study. Individual T1 presented male flowers, whereas the other three trees had female flowers. Fresh leaves from the tree branches were collected in the morning once a month over one year (March/2018 to February/2019) and this material was immediately frozen. Then, a part of the material was lyophilized, ground and stored in hermetically closed flasks at room temperature and in the dark until analysis. A graph presenting the rainfall and temperature during the period of collection is included in the Supplementary Material (Figure S1).

### 2.2. Ethanol Extraction

Dry and ground leaves were weighed (0.4 g) in tubes and 2 mL of absolute ethanol was added. The extraction was performed in a sonic bath for 30 min. Then, the extracts were centrifuged at 8000× *g* for five minutes and the supernatant was transferred to vials. For analytical quality control (QC) a mixture of the same portion of each leaf sample was prepared and extracted following the same steps as the other samples. 

### 2.3. Headspace Solid Phase Microextraction (HS-SPME)

Fresh frozen leaves were ground by mortar and pestle under liquid N_2_ and 0.2 g of each sample was weighed in vials (20 mL). The vial was incubated at 60 °C for 5 min. Then a polydimethylsiloxane/divinylbenzene fiber assembly (PDMS/DVB—65 μm) obtained from Supelco, Bellefonte, PA, USA was exposed in the vial for 5 min at the same temperature. Afterward, the fiber was inserted into the equipment; the whole process was performed automatically. The QC sample was a mixture of equal portions of each sample and was submitted to the same fiber-extraction process as the other samples.

### 2.4. LC-MS Analysis

The analyses were performed using a UPLC Acquity chromatograph coupled to a TQD Acquity Mass spectrometer (Micromass-Waters Manchester, England), with a C18 HSS T3 (2.1 mm × 50.0 mm × 1.8 μm) Waters column, under the following conditions: solvent A (0.1% ammonium hydroxide solution in purified water), solvent B (HPLC grade Methanol); beginning 70% B, ramping to 100% B in 7 min, maintaining until 9 min, then returning to the initial conditions and stabilizing until 12 min. The mass spectra were acquired using electrospray ionization in the negative ion mode with: capillary −3.00 kV, cone −30 V, source temperature of 150 °C and desolvation temperature of 350 °C, full scan between *m*/*z* 100–1000. The concentration of the samples was 1 mg/mL and the volume injected was 4 μL. The analysis of each sample (month/tree) was performed in duplicate and the injections were randomized. The QC samples were injected every seven samples, as well as a blank containing absolute ethanol.

A QC sample was also analyzed on an Agilent Ultra High-Performance Liquid Chromatograph coupled to a high-resolution mass spectrometer, (Agilent 6560 UHPLC-IM-Q-TOF, Santa Clara, CA, USA) for component identification. The analysis was performed with a Jetstream electrospray ion source in negative ion mode, a capillary voltage of −4000 V, and a fragmentor of −400 V; nebulization and sheath gas temperature of 150 °C and 360 °C, respectively. For compound identification, the MS/MS fragmentation was performed with collision energies between 10 and 40 V. The chromatographic separation was carried out on a Waters HSS-T3 2.1 x 50 mm, 1.8 μm column, at 40 °C, flow of 0.4 mL/min. Solvent A was purified water with 0.1% ammonium hydroxide and solvent B was methanol followed the gradient: started with 70% of B, ramping to 100% of B in 7 min, maintaining until 9 min; returning to 70% of B in 9.1 and maintaining this condition until 12 min. 

### 2.5. GC-MS Analysis

These analyses were performed on a Thermo ScientificTM, Waltham, MA, USA 02451. TRACE 1300 gas chromatograph fitted with a HP-5ms fused silica capillary column (Agilent J&W, USA—30 m × 0.25 mm × 0.25 μm film thickness) and coupled to a Thermo Scientific^TM^ ISQ QD mass spectrometer operating in full scan mode (50–450 *m*/*z*). The analytical method was used according to Adams (2007) [12] with modifications: injector temperature 220 °C and oven temperature started at 60 °C increasing to 195 °C at 3 °C/min. The injection was made in split mode with a ratio of 1:20. Helium was used as a carrier gas at 1 mL/min. Some compounds were suggested comparing with literature and the mass spectra with NIST Library from the equipment, and confirmed via Retention Index when possible. The samples were analyzed in duplicate, in addition, QC samples were analyzed at every ten samples.

### 2.6. Data Processing 

Firstly, LC-MS and GC-MS raw data files were converted to the mzXML format using ProteoWizard software, version 3. For the UHPLC-MS data, the preprocessing and parameter optimization were performed using XCMS [13] CAMERA [14] and IPO [15] packages of R (4.0) available at http://www.bioconductor.org/, accessed on 10 March 2020. Final parameters are presented in Table 1.

The XCMS data output was checked using Excel, to exclude redundant mass features, such as in-source fragments and adducts, besides the mass features listed with retention time over 9.1 min, when the chromatographic conditions were stabilizing for a new run. The data were normalized by Support Vector Regression (SVR) using the MetNormalizer packages in R (4.0). Then the chemometric tests were performed using online MetaboAnalyst (https://www.metaboanalyst.ca, version 5.0, accessed on 10 March 2019) [16], wherein the normalized data were mean-centered, followed by the statistical analysis of variance (ANOVA) and Tukey test (*p* < 0.05). Principal component analysis (PCA) was performed to group similar samples and hierarchical cluster analysis (Heatmap) to visualize the impact of variables on the sample groups.

The treatment of GC-MS data was performed in MSdial. The data were normalized by median and the features were auto-scaled via MetaboAnalyst, followed by the statistical analysis of variance (ANOVA) and Tukey test (*p* < 0.05). Principal component analysis (PCA) was performed to group similar samples and hierarchical cluster analysis (Heatmap) to visualize the impact of variables on the sample groups. 

### 2.7. Biological Activity Prediction

Some compounds were putatively identified, followed by Prediction of Activity Spectra for Substances (PASS) software version 2.0, to indicate the possible activity of compounds, mainly volatile compounds. PASS software furnishes information about the biological activity of natural compounds through in silico experiments [17]. In this manner, probable activity (Pa) and probable inactivity (Pi) are estimated.

## 3. Results and Discussion

### 3.1. Non-Volatile Compounds in the Ethanol Extracts and UHPLC-MS

XCMS analysis resulted in a table containing the 110 samples, 161 mass features (*m*/*z* × retention time), and their peak area. After curation, 65 of these mass features were significant enough to separate the samples into distinct groups related to the individual trees. A general analysis including QCs and samples was made initially to verify the analytical instrument variations. The PCA scores graph (PC1 × PC2) showed five distinct groups, with the QCs placed near the center confirming that there were no large variations caused by the extraction and analytical procedures (Figure S2 in Supplementary Material). The QC samples were excluded from further analyses. 

**Figure 1:**
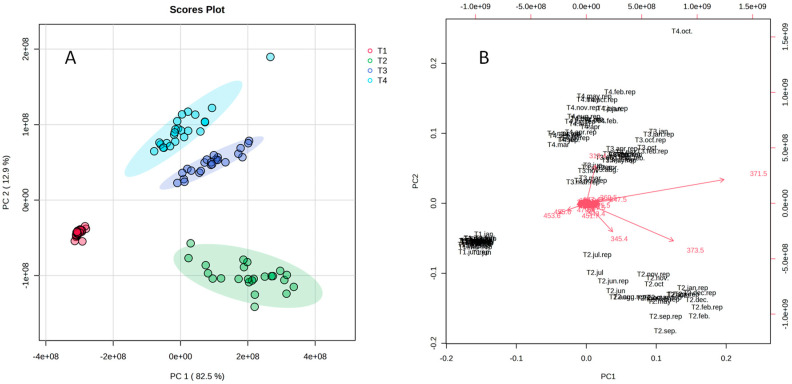
(**A**) PCA score plot (PC1xPC2) of UHPLC-MS analysis of *Schinus terebinthifolia* Raddi leaf extracts and (**B**) PCA biplot of the same extracts.

The PCA scores plot (PC1 × PC2) (Figure 1A) divided the samples into four distinct groups which corresponded to the individual trees throughout the year. In this figure, each circle corresponds to individual injections of each sample and was able to explain a robust 95.4% of the chemical variability. T1 samples were placed far from the other groups in Figure 1A, hence the composition of this individual was the most different from the others, possibly because this is a tree with male flowers. The PCA biplot (Figure 1B) shows the mass features associated with each group: mass features *m*/*z* 453 and 455 were strongly associated with T1. There are some common mass features in T2, T3, and T4 extracts, but with different intensities in each, thus they are grouped separately. T2 presented a higher intensity of *m*/*z* 373 and 345 in the chromatograms whereas *m*/*z* 371 was more intense in T3. Finally, T4 was characterized by a high intensity of *m*/*z* 319. The group of mass features in the center of PCA shows that many mass features are common to all these individuals. No seasonal tendency was observed in these data (Figure S9).

**Figure 2:**
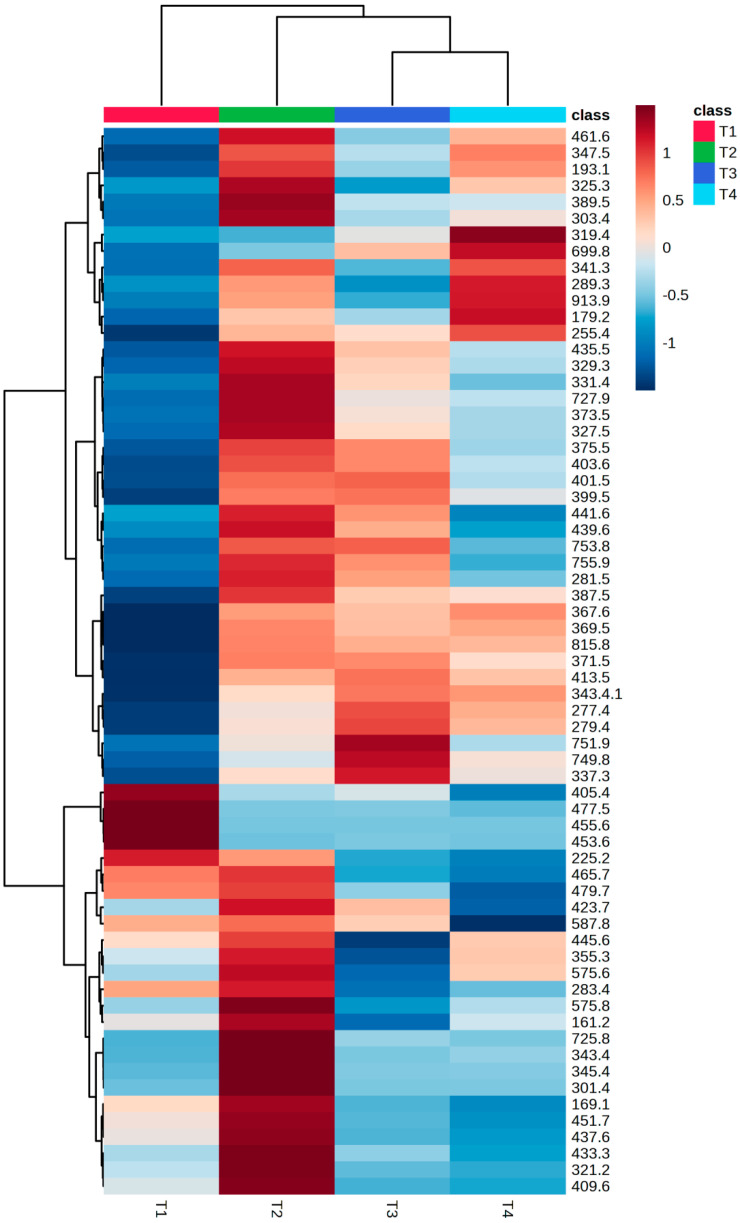
Hierarchical cluster analysis (Heatmap) with top 65 features and groups average of *Schinus terebinthifolia* non-volatiles. Distance: Euclidean. Autoscaling features. Normalized data. Clustering algorithm: Ward.

The heatmap of the same data, now presented as averages for each tree (Figure 2), showed these variations in mass feature intensities more clearly, mainly between T1 and the other plants, just as the PCA. This marked difference can also be observed in the UHPLC-ESI-MS chromatograms (Figure S2 in Supplementary Material), wherein the T1 chromatogram was visibly different from T2, T3, and T4 chromatograms, which present the same peaks, but with different intensities. 

The UHPLC-QTOF analysis resulted in more information about features of interest (Table 2), hence making it possible to propose some compounds’ putative identities. The identification levels were based on Salek et al., [18] considering level 1 identification by comparison to a chemical standard, but levels 2 and 3 are based on comparison to data from the literature.

The extracts contained sugar (*m*/*z* 341 and 179), which is common in leaves and shikimic acid (*m*/*z* 173), and is an important precursor in the phenolic pathway. The MS/MS spectra of *m*/*z* 319, 345, 347, 371, 373, and 375 showed [M–44]^−^ as their main fragment, corresponding to a neutral loss of a carboxyl group (CO_2_). These are probably anacardic acids that have different lengths of alkyl chains and numbers of unsaturations. Their phenol group results in a fragment ion of *m*/*z* 106 and *m*/*z* 119 as a result of the fragmentation in the allyl position of unsaturated acids [19]. Anacardic acids are typical of the Anacardiaceae family, to which *S. terebinthifolia* belongs [20,21]. This class of compounds is described in the literature with antioxidant, antifungal, and anticonvulsive activities [22,23]. 

According to PASS online software, these anacardic acids could also present properties such as taurine dehydrogenase inhibitor, lipoprotein lipase inhibitor, antimutagenic, and cholesterol antagonist, among others. The bioactivity prediction is based on the chemical structure of the compounds; so many compounds with similar structures are associated with the same properties [17]. However, these are interesting activities to verify in future studies.

Mass features *m*/*z* 453 and 455, which were strongly associated with T1, do not have an elucidative fragmentation pattern. However, they were also observed in ethanolic extracts of *S. terebinthifolia* leaves by Gomes et al., [23] with the same mass formula. Masticadienoic acid (*m*/*z* 453) and schinol (*m*/*z* 455) previously described in *S. terebinthifolia* leaf extracts showed antifungal activity against *Paracoccidioides brasiliensis*, anti-inflammatory activity (inhibiting the activity of phospholipase A2) and antileishmanial activity [24,25,26]. 

Finally, features *m*/*z* 183, 197, 335, and 349, were also reported by Gomes et al., [23] and are gallic acid derivatives. Some derivatives of gallic acid were described as responsible for the antioxidant activity of *S. terebinthifolia* extracts [27]. Furthermore, methyl gallate was shown to have antimicrobial activity [28].

Several of these putatively identified components present important biological activities, supporting further studies of this species’s leaves as a source of bioactive molecules. 

**Table 2:** Putatively identified compounds of *Schinus terebinthifolia* ethanol extracts based on high-resolution UHPLC-MS and MS/MS results in comparison to the cited references.

Rt min	[M-H]^−^ (*m*/*z*)	Fragments (*m*/*z*)	Collision Energy (eV)	Proposed Compound	Level	Reference
0.37	341.1111	297.1199; 267.9441; 221.0675; 161.0451; 131.0346; 101.0254; 89.0241; 71.0146; 59.0139	10	Disaccharide C_12_H_21_O_11_	2	[29] metlin
0.50	173.0458	155.0347; 137.0246; 111.0451; 99.0449; 93.0344; 83.0504; 73.0294	10	Shikimic acid C_7_H_10_O_5_	2	[29] metlin
2.48	183.0305	124.0168; 78.0108	20	Methyl gallate C_8_H_8_O_5_	2	[23]
3.23	197.0457	169.0137; 124.0166; 78.0109	20	Ethyl gallate C_9_H_9_O_5_	2	[23]
3.52	335.0418	183.0296; 124.0164; 78.0107	40	Methyl digallate C_15_H_11_O_9_	2	[23]
3.84	349.5650	197.0458; 169.0145; 124.0169	20	Ethyl digallate C_16_H_14_O_9_	2	[23]
4.68	255.2350	-	20	C_16_H_32_O_2_	4	-
4.93	319.2285	275.2385	20	Anacardic acid (13:0) C_20_H_32_O_3_	2	[3]
5.00	369.2473	325.2540; 119.0504	20	C_24_H_34_O_3_	3	-
5.12	453.3373	-	40	Masticadienoic acid C_30_H_46_O_3_	3	[23]
5.13	345.2434	301.2540	20	Anacardic acid (15:1) C_22_H_34_O_3_	2	[19]
5.35	371.2651	327.2694; 133.0664; 119.0503; 106.0430	20	Anacardic acid (17:2) C_24_H_36_O_3_	2	[19]
5.47	455.3549	-	40	Schinol C_30_H_48_O_3_	3	[23]
5.65	347.2648	303.2687; 202.9666; 199.0358; 106.0423	40	Anacardic acid (15:0) C_22_H_36_O_3_	3	[19]
5.79	373.2744	329.2846; 133.0663; 119.0505; 106.0428	40	Anacardic acid (17:1) C_24_H_38_O_3_	2	[19]
6.30	375.2899	331.3007; 119.0502; 106.0425	40	Anacardic acid (17:0) C_24_H_40_O_3_	2	[19]

### 3.2. Volatile Compounds by GC-MS 

The data were preprocessed by MSdial software version 4. and resulted in a table containing 105 samples, 212 mass features, and their respective areas. This table was curated and 37 mass features remained in the table, labeled using the number sequence (1 to 37), but only 31 mass features were significantly different between samples. Twenty-nine mass features were putatively identified based on the NIST library of the GC-MS equipment and the literature (Table 3). 

**Figure 3:**
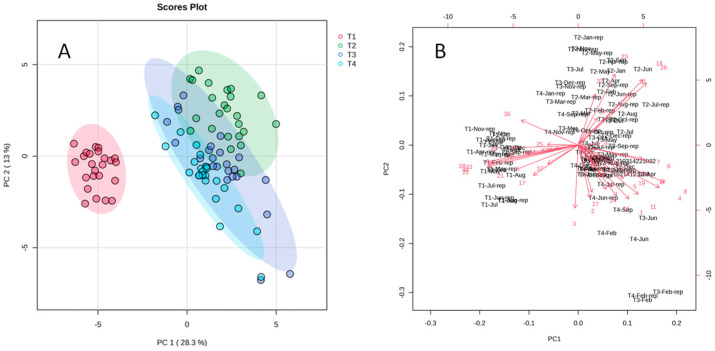
(**A**) PCA score plot (PC1xPC2) of GC-MS analysis of volatiles in the *Schinus terebinthifolia*’ leaves and (**B**) PCA biplot of the same samples.

The PCA scores plot (PC1xPC2) (Figure 3A) explained 28.3% of the variation on the X axis and a further 13% on the Y axis, with a total of 41.3% of explained data variance by PCA. This result shows that the main difference in composition was between T1 and the group formed by T2, T3, and T4. The PCA scores plot with QC samples may be seen in Supplementary Material (Figure S4).

Following the same trend as the non-volatile compounds, T1 samples were grouped on the opposite side of the others whereas T2, T3, and T4 are practically superimposed. The PCA biplot (Figure 3B) indicates one subset of mass features for T1 and a different subset for T2–T4. This can also be observed in the GC-MS chromatograms (Figures S5–S8, Supplementary Material). As T1 was the only male individual, this may explain the marked difference in its volatile leaf composition, confirming that the individual tree was more important than the season of collection (Figure S10). 

**Figure 4:**
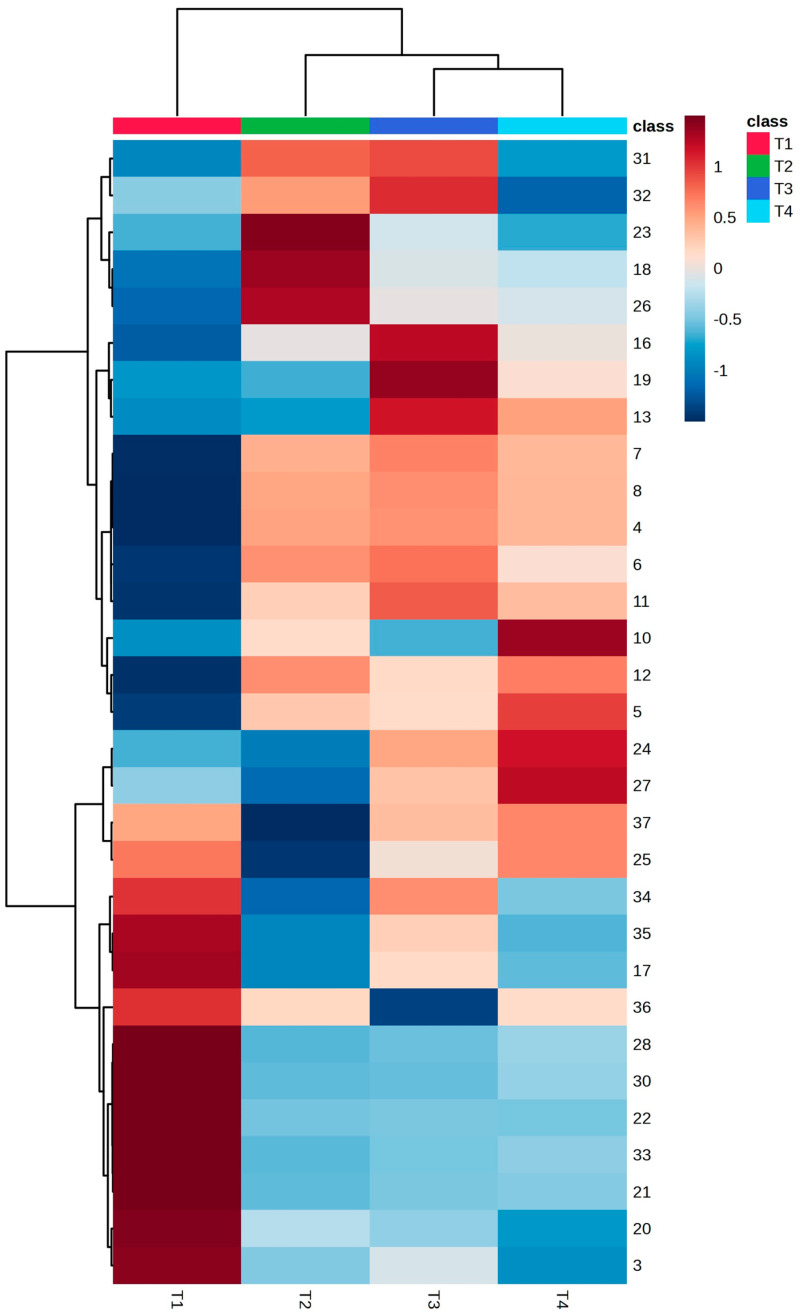
Hierarchical cluster analysis (Heatmap) with top 31 features and groups average of *Schinus terebinthifolia* volatiles. Distance: Euclidean. Autoscaling features. Data were normalized. Clustering algorithm: Ward. Numbered according to Table 3.

The heatmap of the same data now presented as averages for each tree (Figure 4) shows the mass features associated with each group. Samples of T2, T3, and T4 showed some mass features in common, while T1 samples were associated with different mass features.

According to the Heatmap (Figure 4), the sesquiterpenes were more effective in separating the individuals. T1 presented more sesquiterpenes in its composition, and in higher intensity than the other leaf samples, i.e., α-Copaene (19), β-Elemene (22), β-Bourbonene (20), Bicyclogermacrene (33), α-Muurolene (34), γ-Muurolene (29), Alloaromandendrene (28), Germacrene D (30), Caryophyllene (24), Humulene (27) β-Selinene (31), α-Selinene (32) and δ-Cadinene (37); although the monoterpenes, α-Pinene (1) andβ-Pinene (3) were also more intense in T1 samples (Table S1). These compounds have been found in essential oils of other plants, but studies of the biological activities of the isolated compounds are rare. Therefore, the PASS software predictive result was used to indicate possible properties of these compounds (Table 3). 

Despite T2–T4 samples being practically superimposed in the PCA (Figure 3), in the heatmap (Figure 4) it is possible to note the correlation of some compounds with each individual tree. T2 presented the sesquiterpenes: α-Gurjunene (23) and Isoledene (18) in higher intensity, as well as Aromandendrene (26); it presented the highest percentage of the monoterpene 3-Carene (6), as well (Table S2). T3 was characterized by the presence of the sesquiterpenes: α-Copaene (19), β-Selinene (31), and α-Selinene (32) (Table S3). Finally, T4 presented the highest percentage of α-Phellandrene (5) and the sesquiterpenes: Caryophyllene (24) (Table S4).

PASS software predictive result also indicates possible properties for these compounds (Table 3). 

The chemical variations in volatile and non-volatile compounds of *S. terebinthifolia* leaves varied more intensely between individuals and were not strongly affected by seasonal effects in this region of Brazil. Neither PCA nor partial least squares discriminant analysis (PLSDA) plots (Figures S9 and S10 in Supplementary Material) of both volatile and non-volatile components showed any groups formed by months of the year or by seasons. Native trees were selected randomly in this study to represent a realistic variability among individuals and were exposed to the same conditions of rain, light, and temperature during the year due to their geographic proximity. Although the age of the plant, the kind of soil and the availability of nutrients and water are factors that could explain some chemical differences between these individuals, apparently the sex of the tree was the main factor responsible for the variation in composition. T1 had male flowers while the others had female flowers, which indicates that the chemical composition of the leaves may be strongly affected by the sex of the flowers. However, further studies, with a larger number of individuals, could confirm this hypothesis.

## 4. Conclusions

According to this study, leaves from the same *S. terebinthifolia* tree had similar volatile and non-volatile chemical compositions throughout the year. However, the chemical composition and the intensity of compounds varied significantly between trees, especially between the plant with male flowers and the others, growing within a two kilometers radius in Campinas (Brazil). Thus, the choice of the individual is more important than the month of collection for the standardized use of its leaves. Volatile compounds varied less than non-volatile compounds between the individuals. Further studies with a larger number of trees will be necessary to confirm if male and female trees consistently present different chemical compositions. However, for both sexes, chemical compounds with important biological activities, related in the literature and suggested by PASS software, were putatively identified, confirming the use of *S. terebinthifolia* leaves as a source of bioactive compounds; reducing waste and increasing economic gains for local farmers throughout the year.

## Figures and Tables

**Table 1:** XCMS parameters optimized by IPO package in RStudio for analysis of *Schinus terebinthifolia* LC-MS chromatograms.

Feature Detection	
Method	matchedFilter
FWHM	10
step	0.3
Retention Time Correction	
Method	obiwarp
profStep	1
Alignment	
bw	22
minfrac	0.4
mzwid	0.1

**Table 3:** Volatile compounds of *Schinus terebinthifolia* leaves are putatively identified by GC-MS and other references for these compounds in the literature.

Feature Number	R. I. lit	R.I. exp	Compounds	Class	Reference	Biological Properties—Predictive PASS Software
1	939	940	α-Pinene	M	[7]	Phobic disorders treatment and carminative
2	975	974	Sabinene	M	[7]	Antiinflammatory and antineoplastic
3	979	978	β-Pinene	M	[7]	Ovulation inhibitor and cardiovascular analeptic
4	990	994	β-Myrcene	M	[30]	Mucomembranous protector and antineoplastic (breast cancer)
5	1002	1007	α-Phellandrene	M	[7]	Carminative and fibrinolytic
6	1011	1017	3-Carene	M	[7]	Phobic disorders treatment and carminative
7	1026	1030	*p*-Cymene	M	[7]	Antieczematic and carminative
8	1029	1030	D-Limonene	M	[7]	Apoptosis agonist and antineoplastic
9	1050	1049	β-Ocimene	M	[7]	Apoptosis agonist and mucomembrane protector
10	-	1057	Unknown	-	-	-
11	1059	1059	γ-Terpinene	M	[7]	Phobic disorders treatment and carminative
12	1086	1089	Terpinolene	M	[31]	Antieczematic and carminative
13	-	1102	Unknown	-	-	-
14	-	1131	Unknown	-	-	-
15	1177	1177	Terpinen-4-ol	M a	[7]	Antiseborrheic, antieczematic and carminative
16	-	1208	Unknown	-	-	
17	-	1348	Unknown	-		-
18	1374	1368	Isoledene	S	[32]	Transplant rejection treatment
19	1375	1381	α-Copaene	S	[31,33]	carminative and anticancer
20	1387	1379	β-Bourbonene	S	[3]	Anticancer and prostate disorders treatment
21	-	1381	Unknown	-	-	-
22	1390	1396	β-Elemene	S	[7]	Carminative and anti-inflammatory
23	1409	1407	α-Gurjunene	S	[3]	Transplant rejection treatment
24	1419	1420	Caryophyllene	S	[7]	Antineoplastic and anti-inflammatory
25	-	1424	Unknown	-	-	-
26	1441	1440	Aromandendrene	S	[7]	Antineoplastic and antiosteoporosis
27	1454	1451	Humulene	S	[7]	Antineoplastic and anti-inflammatory
28	1460	1460	Alloaromadendrene	S	[7]	Antineoplastic and antieczematic
29	1478	1472	γ-Muurolene	S	[33]	Antieczematic and carminative
30	1481	1481	Germacrene D	S	[7]	Antieczematic and carminative
31	1490	1488	β-Selinene	S	[31]	Antipsoriatic and anti-inflammatory
32	1498	1498	α-Selinene	S	[31]	Carminative, antieczematic, antineoplastic, and apoptosis agonist
33	1500	1501	Bicyclogermacrene	S	[7]	Phobic disorders treatment and as an antieczematic
34	1500	1502	α-Muurolene	S	[33]	Carminative and antineoplastic
35	-	1506	Unknown	-	-	-
36	1513	1519	γ-Cadinene	S	[7]	Antieczematic and carminative
37	1523	1526	δ-Cadinene	S	[7]	Antieczematic and antipsoriatic

M—monoterpene, S—sesquiterpene, Ma—monoterpene alcohol, R.I. lit—retention index according to literature, R.I. exp.—experimental retention index.

## Data Availability

The original contributions presented in the study are included in the article or Supplementary Material, further inquiries can be directed to the corresponding author.

## Supplementary Materials

The following supporting information can be downloaded at: https://www.mdpi.com/article/10.3390/metabo14110612/s1, Figure S1. Monthly rainfall and maximum and minimum temperatures in Campinas, SP between March 2018 and February 2019. Figure S2. (A) PCA score plot (PC1xPC2) of UHPLC-MS analysis of Schinus terebinthifolia Raddi leaf extracts and (B) PCA biplot of the same extracts. Including the QC samples. Figure S3. Typical UHPLC-ESI-MS chromatograms of extracts of the four *Schinus terebinthifolia* individuals. Figure S4. (A) PCA score plot (PC1xPC2) of GC-MS analysis of *Schinus terebinthifolius* Raddi leaf and (B) PCA biplot of the same samples. Including the QC samples. Figure S5. Typical GC-MS chromatogram of leaves of the *Schinus terebinthifolia*—T1. Figure S6. Typical GC-MS chromatogram of leaves of the *Schinus terebinthifolia*—T2. Figure S7. Typical GC-MS chromatogram of leaves of the *Schinus terebinthifolia*—T3. Figure S8. Typical GC-MS chromatogram of leaves of the *Schinus terebinthifolia*—T4. Figure S9. (A) PCA score plot (PC1xPC2) of UHPLC-MS analysis of *Schinus terebinthifolia* leaf extracts, samples marked according to the month of collection and (B) PLSDA score plot of the same extracts. Figure S10. (A) PCA scores plot (PC1xPC2) of the results of the GC-MS analysis of *Schinus terebinthifolia* leaves, samples marked according to the month of collection and (B) PLSDA score plot of the same samples. Table S1. Average (%) and SD of identified volatile compounds of *Schinus terebinthifolia* leaves in T1 over one year. Table S2. Average (%) and SD of identified volatile compounds of *Schinus terebinthifolia* leaves in T2 over one year. Table S3. Average (%) and SD of identified volatile compounds of *Schinus terebinthifolia* leaves in T3 over one year. Table S4. Average (%) and SD of identified volatile compounds of *Schinus terebinthifolia* leaves in T4 over one year.
