# Supplementary material for: Schinus terebinthifolia Raddi—Untargeted Metabolomics Approach to Investigate the Chemical Variation in Volatile and Non-Volatile Compounds
Source: Metabolites. 2024 Nov 11;14(11):612. doi: 10.3390/metabo14110612 (PMC11596854; doi:10.3390/metabo14110612)
Supplement: Supplementary file 1 [file metabolites-14-00612-s001.zip › metabolites-3283845-supplementary.pdf]

Supplementary Material

Schinus terebinthifolia Raddi – Untargeted metabolomics approach to investigate the chemical variation in volatile and non-volatile compounds

Mara Junqueira Carneiro[1], Guilherme Perez Pinheiro[2], Elisa Ribeiro Miranda Antunes[2], Leandro Wang Hantao[3], Thomas Moritz[4], Alexandra Christine Helena Frankland Sawaya[1*]

1. Faculty of Pharmaceutical Science, State University of Campinas, Campinas SP, Brazil

2. Institute of Biology, State University of Campinas, Campinas SP, Brazil

3. Institute of Chemistry, State University of Campinas, Campinas SP, Brazil

4. Department of Forest Genetics and Plant Physiology, Swedish University of Agricultural Sciences, Umeå, Sweden and University of Copenhagen, Copenhagen, Denmark.

*corresponding author : achfsawa@unicamp.br

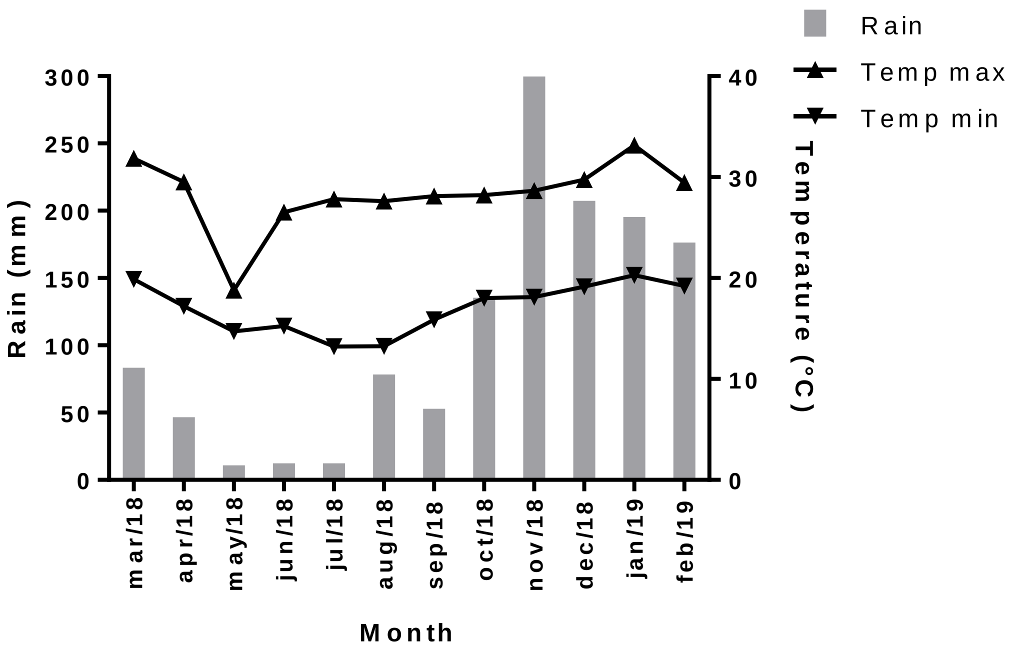

Figure S1. Monthly rainfall and maximum and minimum temperatures in Campinas, SP between March 2018 and February 2019.

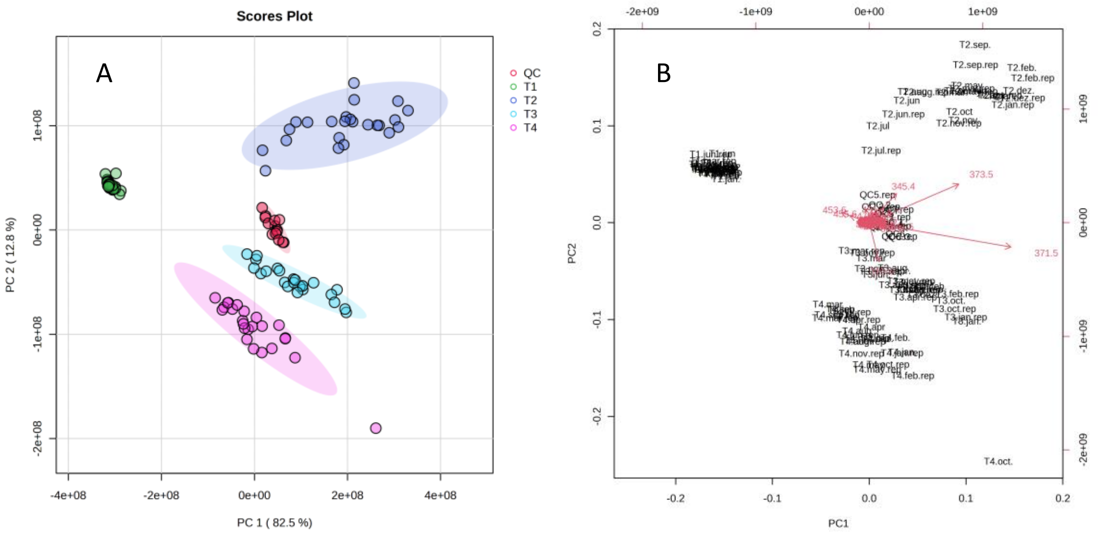

Figure S2. A) PCA score plot (PC1xPC2) of UHPLC-MS analysis of *Schinus terebinthifolia* Raddi leaf extracts and B) PCA biplot of the same extracts. Including the QC samples

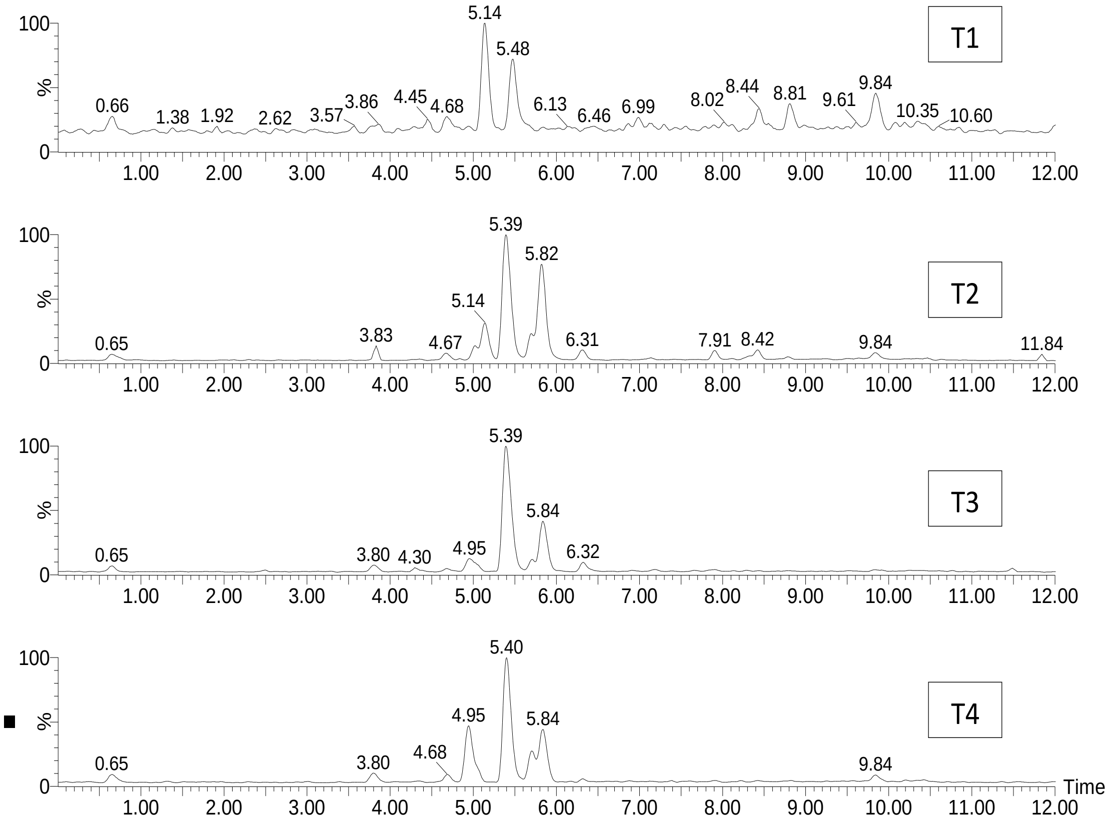

Figure S3. Typical UHPLC-ESI-MS chromatograms of extracts of the four *Schinus terebinthifolia* individuals.

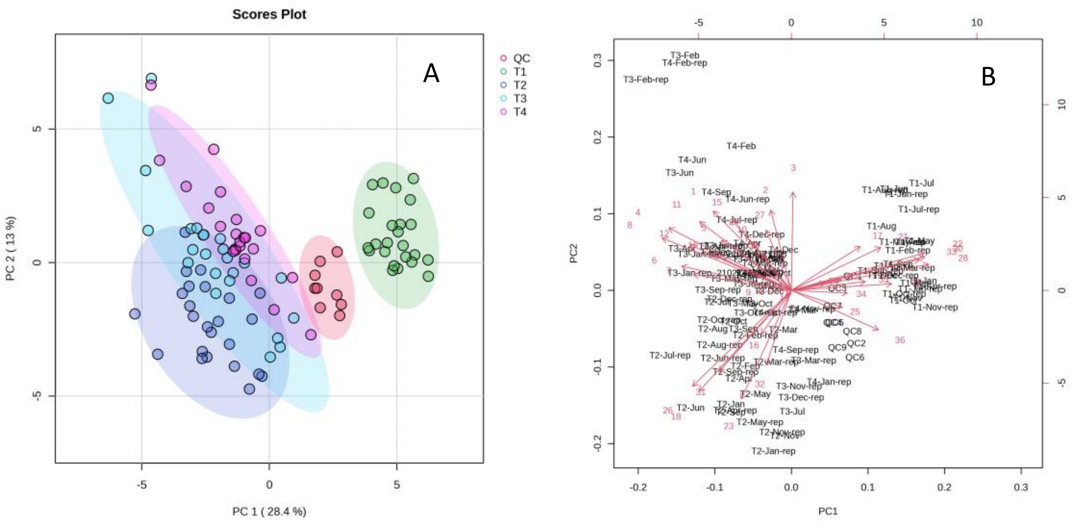

Figure S4. A) PCA score plot (PC1xPC2) of GC-MS analysis of *Schinus terebinthifolius* Raddi leaf and B) PCA biplot of the same samples. Including the QC samples

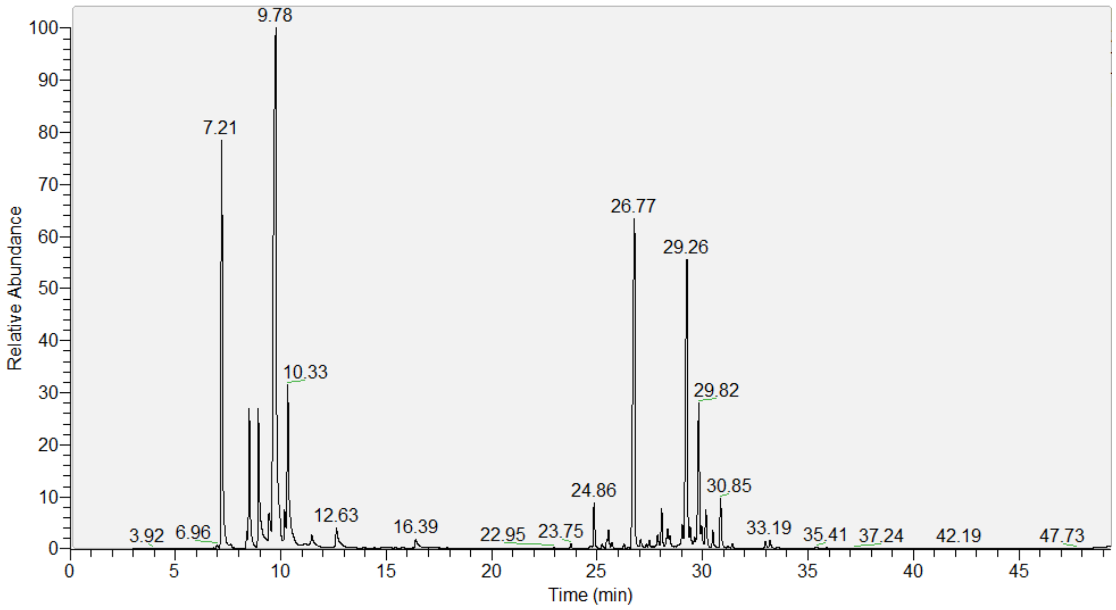

Figure S5. Typical GC-MS chromatogram of leaves of the *Schinus terebinthifolia* – T1

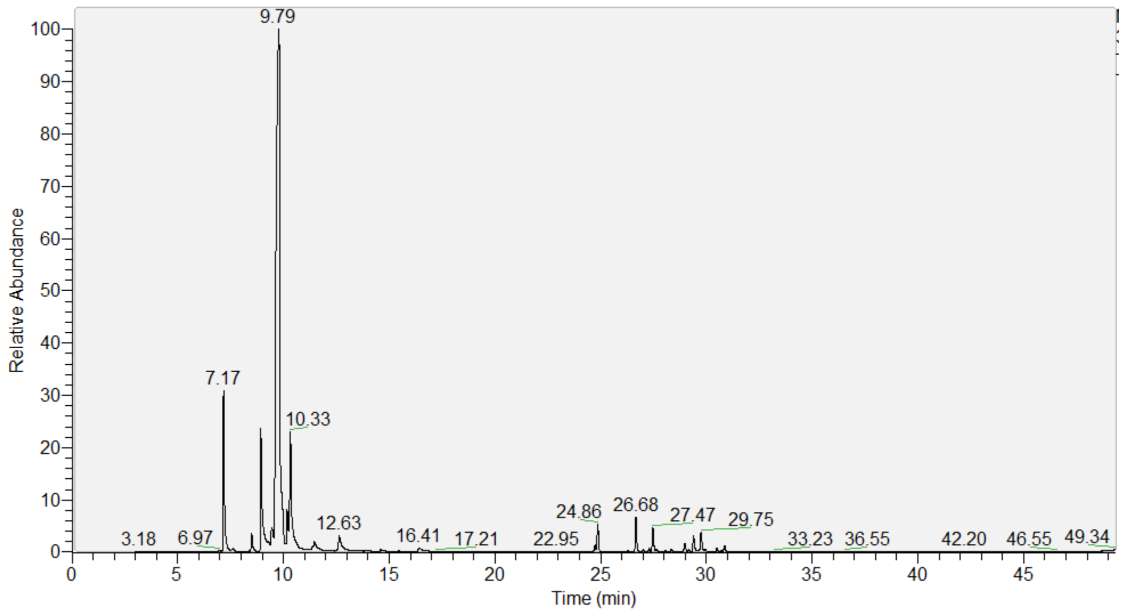

Figure S6. Typical GC-MS chromatogram of leaves of the *Schinus terebinthifolia* – T2

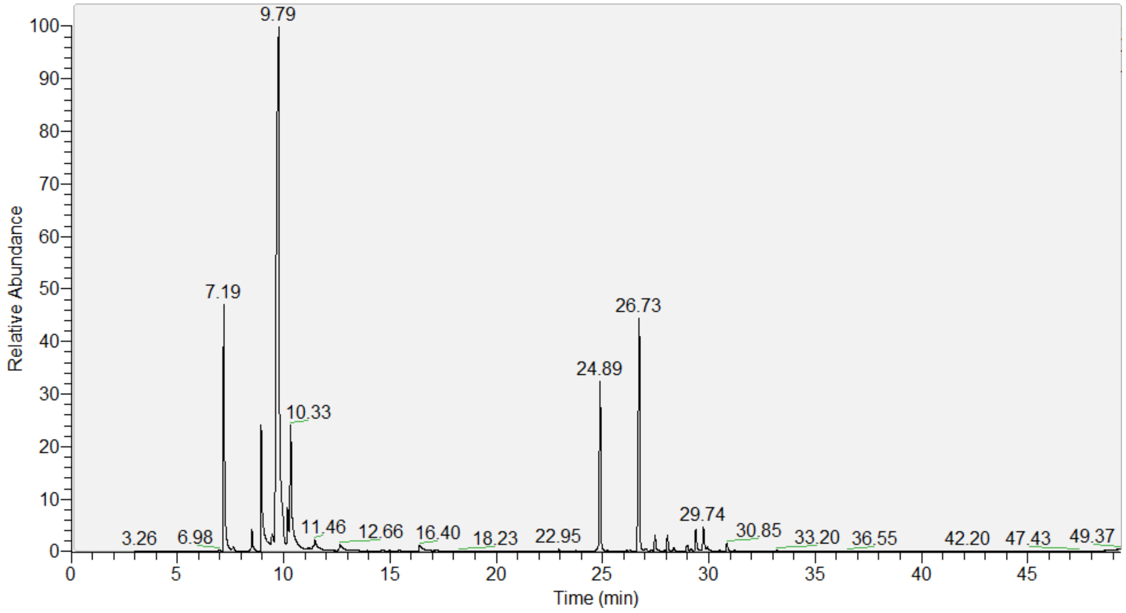

Figure S7. Typical GC-MS chromatogram of leaves of the *Schinus terebinthifolia* – T3

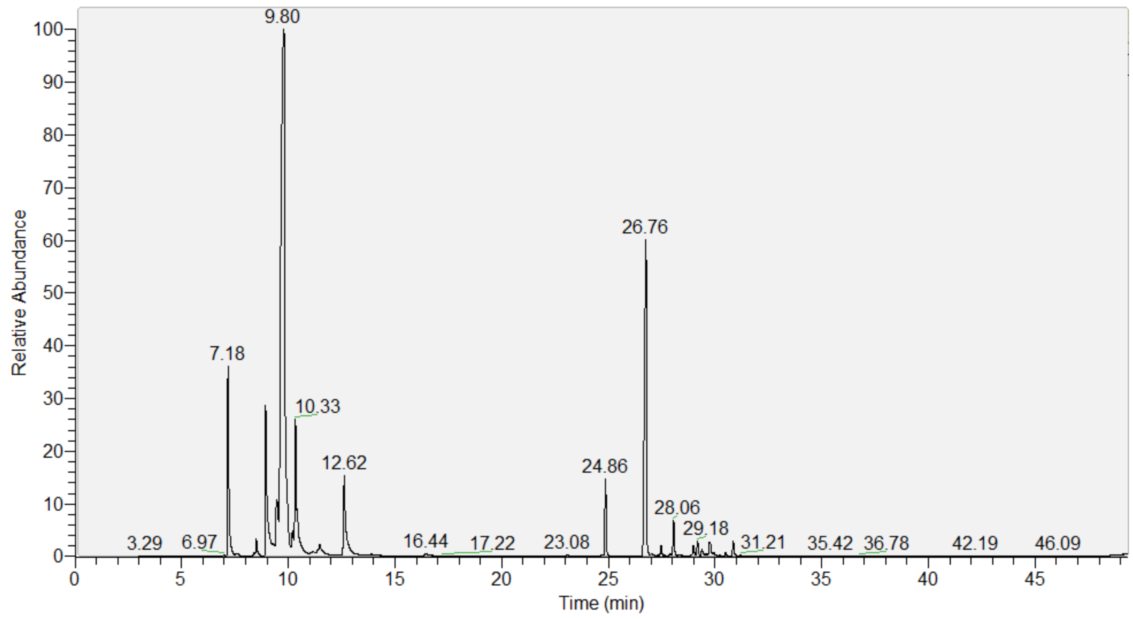

Figure S8. Typical GC-MS chromatogram of leaves of the *Schinus terebinthifolia* - T4

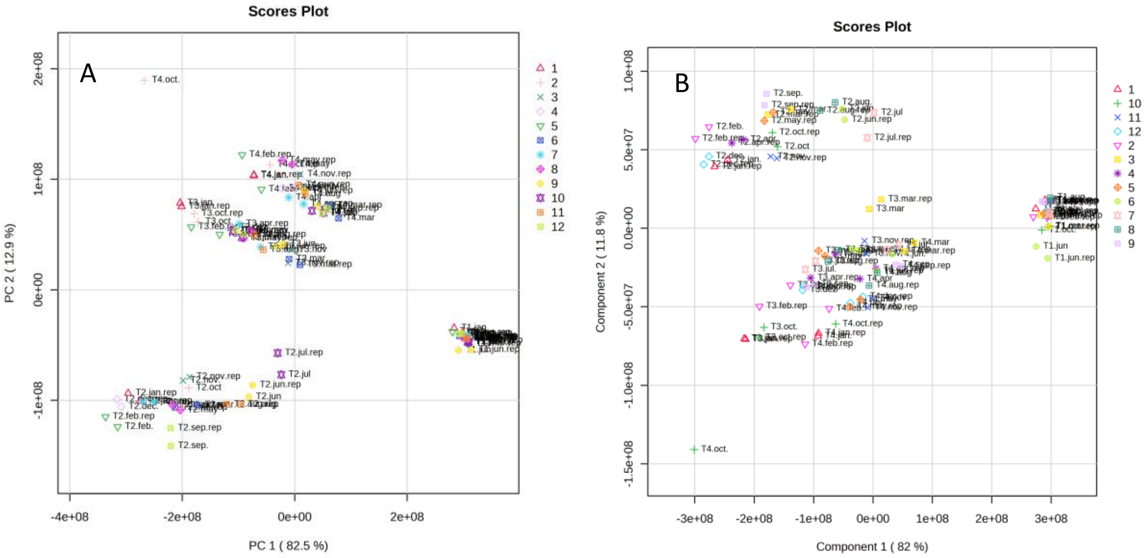

Figure S9. A) PCA score plot (PC1xPC2) of UHPLC-MS analysis of *Schinus terebinthifolia* leaf extracts, samples marked according to the month of collection and B) PLSDA score plot of the same extracts

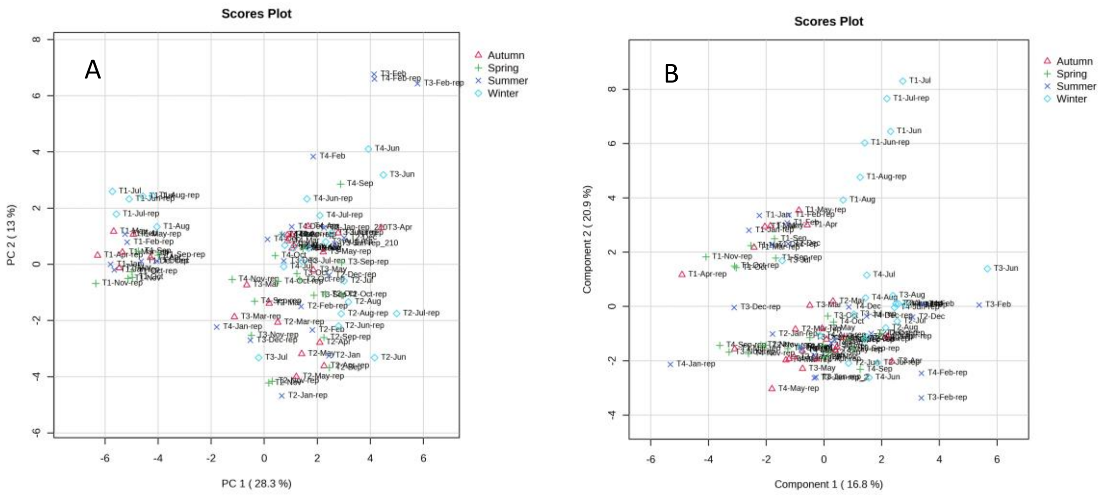

Figure S10. A) PCA scores plot (PC1xPC2) of the results of the GC-MS analysis of *Schinus terebinthifolia* leaves, samples marked according to the month of collection and B) PLSDA score plot of the same samples

Table S1. Average (%) and SD of identified volatile compounds of *Schinus terebinthifolia* leaves in **T1** over one year.

no.	name	average %	SD	T1-Jan	T1-Feb	T1-Mar	T1-Apr-	T1-May	T1-June	T1-July	T1-Aug	T1-Sept	T1-Oct	T1-Nov	T1-Dec
1	α-Pinene	16.5	3.2	15.5	15.1	17.5	17.6	22.2	19.8	17.3	18.3	16.5	15.7	11.9	10.3
2	Sabinene	1.2	0.6	1	1.6	1	0.6	1.1	2	1.1	2.7	0.7	0.9	1	1.4
3	β-Pinene	6.4	1.3	5.8	6.3	6.2	6.6	8.2	8	7.8	7	5.9	6	4.9	3.6
4	β-Myrcene	7.2	1.8	5.7	7.4	6.2	5.4	8.5	8.6	5.5	11.1	7.1	8.5	5	8
5	α-Phellandrene	3.7	1.4	2.8	4.4	3.4	1.7	3.6	5	2.6	6.9	2.8	3.6	3	5
6	3-Carene	25.8	7.8	21	30.3	20.6	31.1	18.5	30	31.5	22.1	35.4	16.6	14.9	38.3
7	p-Cymene	1.4	0.4	1.6	1.2	1.7	1.7	2.1	1	1.2	1.2	1.4	1.9	0.9	1.1
8	D-Limonene	5.5	0.8	5.6	5.3	5.5	5.3	7.1	6.1	4.7	6.6	5.2	5.6	4.7	4.6
9	β-Ocimene	0.1	0.1	0.1	0.1	0	0	0.1	0.2	0.1	0.2	0.1	0.2	0.1	0.1
10	Unknown	0.2	0.2	0	0.1	0.3	0	0.1	0.2	0.2	0.3	0.4	0.6	0	0.2
11	γ-Terpinene	0.6	0.2	0.6	0.5	0.7	0.6	0.9	0.3	0.5	0.4	0.7	1.1	0.6	0.6
12	Terpinolene	2	0.7	2.1	2.4	1.9	1.2	1.7	2.1	1.1	3.5	1.3	1.7	1.8	2.6
13	Unknown	0.3	0.5	0.9	0.1	0.1	0.8	0	0.1	0	0.1	0	0	0	1.6
14	Unknown	0	0	0.1	0	0	0	0.1	0	0.1	0	0	0	0	0
15	Terpinen-4-ol	0.1	0.1	0	0.1	0.2	0	0.2	0.1	0.2	0.1	0.1	0.2	0.2	0
16	Unknown	0	0	0.1	0	0.1	0	0	0	0	0	0	0.1	0	0
17	Unknown	0.2	0.2	0.2	0.1	0.2	0.1	0.1	0.3	0.9	0.1	0.1	0.2	0.2	0.1
18	Isoledene	0	0	0.1	0	0	0	0	0	0	0	0	0.1	0.1	0
19	α-Copaene	1.1	0.5	1.4	0.6	1.3	0.9	0.9	0.8	2	0.6	0.8	1.7	1.5	0.9
20	β-Bourbonene	0.2	0.1	0.3	0.2	0.3	0.2	0.3	0.2	0.2	0.2	0.1	0.2	0.2	0.1
21	Unknown	0.6	0.7	0.6	0.3	0.5	0.2	0.3	0.8	2.6	0.2	0.3	0.8	0.8	0.4
22	β-Elemene	0.4	0.1	0.4	0.3	0.4	0.4	0.4	0.3	0.6	0.3	0.2	0.4	0.4	0.2
23	α-Gurjunene	0.1	0	0.1	0.1	0.1	0.1	0.1	0	0	0	0.1	0.1	0.1	0.1
24	Caryophyllene	5	1.5	7	5	6.7	4.5	5.1	3.3	3.8	3.2	4.6	4.8	8.1	4.4
25	Unknown	0.3	0.1	0.4	0.2	0.4	0.4	0.3	0.1	0.2	0.1	0.2	0.6	0.5	0.2

26	Aromandendrene	0.1	0.1	0.2	0.1	0.2	0.1	0.1	0.1	0	0.1	0.1	0.2	0.2	0.1
27	Humulene	1.4	0.6	1.8	1.2	1.6	1.1	1.1	0.8	1.1	0.7	1.1	2.6	2.4	1
28	Alloaromadendrene	0.5	0.2	0.7	0.4	0.6	0.4	0.4	0.3	0.3	0.2	0.4	0.9	0.9	0.4
29	γ-Muurolene	1.7	1.8	0.6	0.4	0.7	4.5	0.3	0.3	3.5	3.5	0.4	0.4	4.8	0.4
30	Germacrene D	9.9	3.5	14	10.1	13	7.9	10	5.4	6.8	6.5	7.8	12.3	17.1	8
31	β-Selinene	0.3	0.1	0.4	0.2	0.4	0.3	0.3	0.2	0.3	0.2	0.2	0.4	0.4	0.2
32	α-Selinene	0.6	0.3	1	0.3	0.6	0.5	0.3	0.4	0.3	0.4	0.5	1.2	1.3	0.6
33	Bicyclogermacrene	3.7	1.8	5.2	3.6	4.7	3	3.2	1.8	1.4	2.2	2.9	6	7.3	3.3
34	α-Muurolene	0.5	0.2	0.6	0.3	0.5	0.8	0.4	0.2	0.4	0.2	0.4	0.9	0.8	0.4
35	Unknown	0.3	0.1	0.3	0.2	0.3	0.2	0.2	0.1	0.2	0.1	0.2	0.5	0.5	0.2
36	γ-Cadinene	0.5	0.3	0.7	0.4	0.7	0.4	0.5	0.2	0.3	0.2	0.5	0.9	1.1	0.4
37	δ-Cadinene	1.2	0.5	1.4	0.8	1.3	1.1	1	0.6	1	0.5	1.1	2.1	2.1	0.9

Table S2. Average (%) and SD of identified volatile compounds of *Schinus terebinthifolia* leaves in **T2** over one year.

no.	name	average %	SD	T2-Jan	T2-Feb	T2-March	T2-Apr	T2-May	T2-June	T2-July	T2-Aug	T2-Sept	T2-Oct	T2-Nov	T2-Dec
1	α-Pinene	7.2	2.3	6.4	7.5	4.9	8.1	6.5	7.4	8.9	7	10.5	8.5	1.6	8.7
2	Sabinene	0.2	0.3	0.1	0.1	0.2	0	0.3	0	0.1	0.3	0.1	0.3	0	0.9
3	β-Pinene	1.6	0.4	1.5	1.7	1.2	1.5	1.5	1.6	2	1.6	1.6	2	0.6	2.4
4	β-Myrcene	10.6	1.9	11.8	11.5	8.4	10.8	9.1	10.7	11.4	11	12.1	11.6	6	13.1
5	α-Phellandrene	5.2	1.9	2.3	5.1	5.3	4.9	5.8	4.1	6.9	6.5	4.5	6.6	2.1	8.7
6	3-Carene	54.8	5	52	57.4	57.1	54.5	50.5	60.9	51.6	59.1	53	53.3	62.9	45.1
7	*p*-Cymene	2.4	0.8	2	1.7	2.3	2.6	4	2	2.3	1.3	2.6	1.8	3.5	2
8	D-Limonene	7.1	1	8.2	6.1	6.2	7.1	8.8	6	7.4	6	7	6.7	7.4	8.5
9	β-Ocimene	0.2	0.2	0.1	0.2	0	0.6	0.5	0.1	0.1	0.1	0.1	0.1	0	0.1

10	Unknown	0.3	0.2	0.1	0.5	0	0.5	0.1	0.5	0.5	0.1	0.3	0.5	0	0.1
11	γ-Terpinene	1.6	0.3	1.9	1.6	1.7	1.6	1.3	1.4	1.6	1.2	1.3	1.7	2.3	1.6
12	Terpinolene	3.2	1	4.9	2.7	3.4	2.4	4.1	2.1	3.8	3.1	2.5	3.3	1.3	4.6
13	Unknown	0.2	0.5	0.1	0.1	2	0	0.1	0	0	0.1	0	0.1	0.1	0.1
14	Unknown	0.1	0.2	0	0	0	0.6	0.5	0	0	0	0	0	0	0
15	Terpinen-4-ol	0.2	0.1	0.2	0.3	0.1	0.2	0.2	0.2	0.2	0.2	0.1	0.2	0.5	0.1
16	Unknown	0.2	0.2	0.5	0.1	0.3	0.1	0.2	0	0.1	0.1	0	0.1	0.6	0.1
17	Unknown	0	0	0	0	0	0	0	0	0	0	0	0	0.1	0
18	Isoledene	0.2	0.1	0.3	0.2	0.1	0.3	0.3	0.2	0.1	0.1	0.2	0.1	0.5	0.1
19	α-Copaene	0.8	0.4	1.3	0.4	1	0.8	1	0.5	0.5	0.4	0.9	0.6	1.7	0.7
20	β-Bourbonene	0	0	0	0	0.1	0	0.1	0	0	0	0	0	0.1	0
21	Unknown	0	0	0	0	0	0	0	0	0	0	0	0	0	0
22	β-Elemene	0	0	0	0	0	0	0	0	0	0	0	0	0	0
23	α-Gurjunene	0.1	0	0.1	0	0	0.1	0.1	0	0	0	0.1	0	0.1	0
24	Caryophyllene	1.2	0.7	1.9	0.7	2.3	0.8	1.3	0.5	0.7	0.5	0.6	0.7	2.6	1.1
25	Unknown	0.1	0.1	0.1	0.1	0.1	0	0.1	0	0.1	0	0	0.1	0.3	0.1
26	Aromandendrene	0.4	0.2	0.6	0.3	0.2	0.4	0.5	0.3	0.2	0.2	0.4	0.2	0.8	0.2
27	Humulene	0.2	0.1	0.3	0.1	0.3	0.1	0.2	0.1	0.1	0.1	0.1	0.1	0.4	0.1
28	Alloaromadendrene	0	0	0	0	0.1	0	0	0	0	0	0	0	0.1	0
29	γ-Muurolene	0.3	0.2	0.5	0.2	0.3	0.3	0.4	0.2	0.2	0.1	0.3	0.2	0.8	0.2
30	Germacrene D	0.3	0.2	0.5	0.2	0.8	0.1	0.4	0.1	0.1	0.2	0.1	0.2	0.4	0.5
31	β-Selinene	0.2	0.1	0.3	0.2	0.2	0.2	0.3	0.2	0.2	0.1	0.3	0.2	0.5	0.1
32	α-Selinene	0.3	0.2	0.6	0.3	0.3	0.4	0.6	0.3	0.3	0.2	0.4	0.1	0.7	0
33	Bicyclogermacrene	0.1	0.1	0	0	0	0.2	0.1	0.1	0.1	0	0.2	0.2	0.3	0.3
34	α-Muurolene	0.1	0.1	0.2	0.2	0.1	0.1	0.2	0.1	0.1	0.1	0.1	0.1	0.3	0.1
35	Unknown	0	0	0.1	0	0.1	0	0.1	0	0	0	0	0	0.1	0
36	γ-Cadinene	0.2	0.1	0.3	0.1	0.2	0.2	0.3	0.1	0.1	0.1	0.1	0.1	0.4	0.1
37	δ-Cadinene	0.3	0.2	0.5	0.2	0.4	0.3	0.5	0.2	0.2	0.2	0.3	0.2	0.7	0.2

Table S3. Average (%) and SD of identified volatile compounds of *Schinus terebinthifolia* leaves in **T3** over one year.

no.	name	average %	SD	T3-Jan	T3-Feb	T3-March	T3-Apr	T3-May	T3-June	T3-July	T3-Aug	T3-Sept	T3-Oct	T3-Nov	T3-Dec
1	α-Pinene	8.1	2	11.2	7.7	8.4	11.3	9.1	7.6	5.6	6.3	8.4	9.9	6.6	5.6
2	Sabinene	0.7	0.8	0.9	3	0.7	0.1	0	0.7	0.2	0.2	0.7	1.2	0.1	0.1
3	β-Pinene	1.7	0.4	1.9	2.1	2.2	1.8	1	1.8	1.3	1.5	1.6	2	1.4	1.3
4	β-Myrcene	10.1	2.3	6.4	11.2	15.2	12.3	8.3	10.6	8.3	10.7	10	11.3	8.6	8.4
5	α-Phellandrene	5.1	2.1	0.8	4.9	9.7	4.7	4	7	3.9	5.9	4.8	6.2	5.1	4.2
6	3-Carene	46.4	14.2	50.8	46.8	4.8	48.3	56.2	48.6	56.3	54.4	49.1	36	52.8	52.5
7	*p-Cymene*	2.2	1.1	4.5	2	4.7	2.1	1.9	1.1	1.9	1.4	2	2.1	1.4	1.9
8	D-Limonene	6.7	1.8	7.4	6.5	11.9	7.4	5.6	6	5.7	5.5	6.4	7.2	5.7	5.8
9	β-Ocimene	0.1	0.2	0.6	0	0.1	0.1	0	0.1	0.1	0.2	0.1	0.1	0	0
10	Unknown	0.1	0.2	0	0	0.1	0.5	0	0.1	0.1	0.4	0.1	0	0	0.1
11	γ-Terpinene	1.8	1	1.2	4.4	3.2	1.6	1.2	1	1.2	1.2	1.7	1.5	1.5	1.4
12	Terpinolene	2.8	1.8	0	1.9	7.5	2.3	2.1	4	1.9	3.2	2.6	3.4	2.8	2.5
13	Unknown	1.2	1.7	0	0.2	4.6	0.3	0	4	0.1	3.3	1.1	0	0.1	0
14	Unknown	0	0	0	0	0	0	0	0	0	0	0	0	0	0
15	Terpinen-4-ol	0.3	0.4	0.3	1.4	0.2	0.3	0.2	0.1	0.3	0.1	0.4	0.2	0.2	0.3
16	Unknown	0.3	0.2	0.1	0.1	0.7	0.1	0.1	0.2	0.8	0.2	0.2	0.2	0.1	0.4
17	Unknown	0.1	0	0	0	0.1	0	0	0	0.1	0	0	0.1	0	0.1
18	Isoledene	0.1	0.1	0.1	0	0.3	0.1	0.1	0	0.1	0	0.1	0.1	0.1	0.1
19	α-Copaene	4.1	1.4	5.9	3.1	5.8	2.5	3.5	3	4.4	1.9	3.8	6.4	4.6	4.7
20	β-Bourbonene	0	0	0	0	0.1	0	0	0	0.1	0	0	0	0	0
21	Unknown	0	0	0	0	0.1	0	0	0	0	0	0	0	0	0
22	β-Elemene	0	0	0	0	0	0	0	0	0	0	0	0	0	0
23	α-Gurjunene	0	0	0	0	0.1	0	0	0	0	0	0	0	0	0
24	Caryophyllene	4.6	2	5.3	3.2	8.5	2.6	4	2.4	4.6	2	4	7.3	5	6.1
25	Unknown	0.1	0.1	0.1	0	0.4	0.1	0.1	0	0.1	0	0.1	0.1	0.1	0.2

26	Aromandendrene	0.2	0.1	0.2	0.1	0.4	0.1	0.1	0.1	0.2	0.1	0.1	0.2	0.2	0.2
27	Humulene	0.8	0.4	0.8	0.6	1.5	0.4	0.8	0.4	0.8	0.3	0.7	1.4	0.9	1.1
28	Alloaromadendrene	0	0.1	0	0	0.2	0	0	0	0	0	0	0	0	0.1
29	γ-Muurolene	0.2	0.2	0.2	0.1	0.9	0.1	0.2	0.1	0.2	0.1	0.2	0.3	0.2	0.3
30	Germacrene D	0.5	0.9	0.1	0.1	3.2	0.1	0.1	0.1	0.2	0.2	0.3	0.6	0.4	0.7
31	β-Selinene	0.2	0.1	0.2	0.1	0.5	0.1	0.2	0.1	0.3	0.1	0.2	0.3	0.3	0.2
32	α-Selinene	0.3	0.2	0.2	0.1	0.7	0.2	0.3	0.2	0.4	0.2	0.3	0.5	0.4	0.4
33	Bicyclogermacrene	0.2	0.1	0.1	0.1	0.4	0.1	0.1	0.1	0.2	0.1	0.2	0.4	0.2	0.2
34	α-Muurolene	0.2	0.2	0.1	0.1	0.6	0.1	0.1	0.1	0.2	0.1	0.1	0.2	0.2	0.2
35	Unknown	0.1	0.1	0.1	0	0.3	0	0.1	0	0.1	0	0.1	0	0.2	0.1
36	γ-Cadinene	0.1	0.1	0.1	0	0.4	0	0.1	0	0.1	0	0.1	0.1	0.1	0.1
37	δ-Cadinene	0.4	0.3	0.3	0.2	1.3	0.2	0.4	0.2	0.4	0.2	0.4	0.6	0.5	0.7

Table S4- Average (%) and SD of identified volatile compounds of *Schinus terebinthifolia* leaves in **T4** over one year.

no	name	average%	SD	T4-Jan	T4-Feb	T4-March	T4-Apr	T4-May	T4-June	T4-July	T4-Aug	T4-Sept	T4-Oct	T4-Nov	T4-Dec
1	α-Pinene	8.4	2.1	5.1	7.3	7.7	10.4	8.5	12.2	10.4	10.4	5.5	8.3	7.2	7.3
2	Sabinene	0.5	0.8	0.1	2.9	0	0.1	0.1	0.4	1	0.3	0.1	0.1	0.1	0.3
3	β-Pinene	1.5	0.5	1	1.9	1.1	1.8	1.4	2	2.3	2.4	0.6	1.4	1.2	1.5
4	β-Myrcene	10.5	3.2	7.8	12.7	10.3	13.6	8.2	16.7	10.1	14.3	6.1	7.3	8.6	10.1
5	α-Phellandrene	6.8	4.9	5	7.5	4.9	6.5	3.7	7.8	5.7	3.8	21.7	4.4	4.9	5.8
6	3-Carene	43.7	7.6	51.6	36.9	47.3	37.2	43.9	26.2	44	41.1	46.2	53.2	46.4	50.6
7	p-Cymene	2.1	1.3	1.2	1.3	2.4	2.2	5.6	2.2	2.8	2.1	0.8	2.3	1.4	1.4
8	D-Limonene	6.8	1.5	5	7.1	6.9	8	7.9	9.2	7.6	8.3	4.3	5.8	5.2	5.6
9	β-Ocimene	0.1	0.1	0	0.1	0.1	0	0.1	0.1	0.1	0.1	0.1	0.1	0.5	0.1
10	Unknown	0.5	0.7	0	2.5	0.6	0.1	0.4	0.5	0.1	0.5	0	0.6	0	0.7

11	γ-Terpinene	1.6	1	1.1	4.6	2	1.4	1.1	1.7	1	1.7	0.6	1.1	1.3	1.3
12	Terpinolene	3.4	0.8	3.2	3.6	3.2	4.1	2.5	4.9	3.1	4.5	2.2	2.6	3.6	3.1
13	Unknown	0.9	1.3	0.1	0.2	0	1.9	0.1	3.6	0.2	0.2	0	0.1	1.5	3.2
14	Unknown	0.3	1.1	0	0	0	0	0	0	3.7	0	0	0	0	0
15	Terpinen-4-ol	0.4	0.6	0.3	2.2	0.4	0.3	0.3	0.3	0.2	0.3	0.1	0.4	0.2	0.2
16	Unknown	0.1	0.1	0.2	0.1	0.2	0.1	0.2	0.1	0.1	0.2	0.2	0.1	0.1	0.1
17	Unknown	0	0	0	0	0	0	0	0	0	0	0	0	0	0
18	Isoledene	0.1	0	0.1	0	0.1	0.1	0.1	0.1	0.1	0.1	0.1	0.1	0.1	0
19	α-Copaene	2.1	0.6	2	1.8	2.1	2.7	2.9	2.3	1	1.3	2.1	2	3.1	1.5
20	β-Bourbonene	0	0	0	0	0	0	0	0	0	0	0	0	0	0
21	Unknown	0	0	0	0	0	0	0	0	0	0	0	0	0	0
22	β-Elemene	0	0	0	0	0	0	0	0	0	0	0	0	0	0
23	α-Gurjunene	0	0	0	0	0	0	0	0	0	0	0	0	0	0
24	Caryophyllene	6.3	1.9	9.7	4.8	6.6	5.9	8.7	6.8	3.9	4.5	6.1	6	8.7	4.3
25	Unknown	0.1	0	0.2	0	0.1	0.1	0.1	0.1	0.1	0.1	0.1	0.1	0.2	0.1
26	Aromandendrene	0.2	0.1	0.3	0.1	0.1	0.2	0.2	0.1	0.1	0.1	0.1	0.2	0.2	0.1
27	Humulene	1.3	0.4	2	1	1.3	1.2	1.7	1.3	0.7	0.9	1.3	1.2	1.8	0.9
28	Alloaromadendrene	0	0	0.1	0	0.1	0	0	0	0	0.1	0	0	0.1	0
29	γ-Muurolene	0.3	0.1	0.6	0.2	0.4	0.3	0.4	0.3	0.3	0.3	0.3	0.4	0.5	0.2
30	Germacrene D	0.6	0.2	0.9	0.4	0.8	0.5	0.4	0.3	0.4	0.8	0.3	0.7	1.1	0.8
31	β-Selinene	0.1	0	0.2	0	0.1	0.1	0.1	0.1	0.1	0.2	0.1	0.2	0.2	0.1
32	α-Selinene	0.2	0.1	0.2	0	0.2	0.2	0.2	0.1	0.2	0.2	0.2	0.3	0.3	0.1
33	Bicyclogermacrene	0.2	0.1	0.5	0.1	0.2	0.3	0.2	0.1	0.1	0.3	0.2	0.3	0.4	0.2
34	α-Muurolene	0.1	0	0.2	0.1	0.1	0.1	0.2	0.1	0.1	0.1	0.1	0.1	0.2	0.1
35	Unknown	0.1	0	0.1	0	0.1	0	0.1	0	0	0	0	0	0.2	0
36	γ-Cadinene	0.1	0.1	0.3	0.1	0.2	0.1	0.2	0.1	0.1	0.1	0.1	0.2	0.2	0.1
37	δ-Cadinene	0.4	0.1	0.7	0.3	0.5	0.4	0.5	0.3	0.2	0.4	0.4	0.4	0.6	0.3
